# Navigating Emergency Management of Cancer Patients: A Retrospective Study on First-Time, End-Stage, and Other Established Diagnoses in a High Turnover Emergency County Hospital

**DOI:** 10.3390/medicina61010133

**Published:** 2025-01-15

**Authors:** Mihaela Corlade-Andrei, Radu-Alexandru Iacobescu, Viorica Popa, Alexandra Hauta, Paul Nedelea, Gabriela Grigorasi, Monica Puticiu, Roxana Elena Ciuntu, Andreea Ivona Sova, Diana Cimpoesu

**Affiliations:** 1 Department of Surgery II, Emergency Medicine, University of Medicine and Pharmacy “Grigore. T. Popa”, 700115 Iasi, Romania; mihaela.corlade2@umfiasi.ro (M.C.-A.); alexandra.hauta@umfiasi.ro (A.H.); paul-lucian.nedelea@umfiasi.ro (P.N.); roxana-elena.ciuntu@umfiasi.ro (R.E.C.); dcimpoiesu@yahoo.com (D.C.); 2 Emergency Care Department, Emergency “St. Spiridon” Hospital, 700111 Iasi, Romania; gabriela.tiuica@yahoo.com; 3 Department of Medicine II, Nursing, University of Medicine and Pharmacy “Grigore. T. Popa”, 700115 Iasi, Romania; andreea-ivona_sova@umfiasi.ro; 4 Department of Emergency Medicine, West University “Vasile Goldis”, 310025 Arad, Romania; puticiumonica@yahoo.com

**Keywords:** cancer, neoplasia, oncologic, malignancies, emergency department

## Abstract

*Background and Objectives*: The incidence and prevalence of cancers are increasing worldwide, with special emphasis placed on prevention, early detection, and the development of new therapeutic strategies that strongly impact patient outcomes. Limited data are available about emergency care’s role in treating patients with cancer. This study aims to determine the burden of end-stage and first-time diagnosis of cancer on emergency care in a high-turnover emergency care center. *Materials and Methods*: A retrospective observational study was conducted to identify patients requesting emergent aid for cancer-related symptoms in the Emergency Department of “St. Spiridon” Hospital from Iasi (Romania) between 1 October 2022 and 30 September 2023. The proportion and demographic characteristics of end-stage patients and those who received a first-time diagnosis during the emergency visit were evaluated. Risk analysis was performed to understand these patients’ care needs (such as medical care, surgical care, specialty consults, intensive care, ward admission, and other hospital transfers) and immediate care outcomes (such as in-hospital mortality and home discharge). *Results*: 2318 patients with cancer requested emergent care (patient presentation rate of 3.08%), of which 444 (19.15%) were diagnosed for the first time, and 616 (26.57%) were at the end-stage. First-time diagnosed patients had a significantly different distribution of cancer types (*p* < 0.001), were more likely to need any form of medical care, to require a specialty consultation, or to be admitted to a ward (OR 2.65, 95% CI: 2.12–3.32; *p* < 0.001; OR 3.28, 95% CI: 2.48–4.35, *p* < 0.001; OR 2.09, 95% CI: 1.70–2.59; *p* < 0.001, respectively) but were less severe, while end-stage patients were more likely to address repeatedly to the emergency room (OR 1.86, 95% CI: 1.32–2.59; *p* = 0.001) and had higher odds of needing intensive care, assisted ventilation and death (OR-4.63, 95% CI: 1.10–19.45, *p* = 0.04; OR 2.59, 95% CI: 1.57–4.28; *p* < 0.001, and OR 4.06, 95% CI: 1.73–9.54; *p* = 0.001, respectively). Conclusions: The emergency department often carries the weight of diagnosing patients with cancer and treating patients with end-stage disease. These data highlight the importance of prehospital care, particularly for cancer screening and palliative care, and the importance of fostering multidisciplinary collaboration in the emergency room with oncologists, geriatricians, and palliative care specialists to improve patient outcomes.

## 1. Introduction

Cancer is becoming an ever greater health concern as demographic-based predictions indicate that the number of new cases will reach 35 million by 2050 [1]. Due to the increasing prevalence of cancer and improved survival rates, the number of oncological patients visiting the emergency department (ED) may increase [2,3]. Annually, 4 million visits to EDs and other oncology urgent care centers are generated by patients with advanced-stage cancers [4]. These data hint at emergency care’s important role in end-of-life and palliative care [5]. Furthermore, approximately 40% of all men and women in the United States will be diagnosed with a malignancy during their lifetime, with the emergency department being an essential site for the care of these patients [6]. Thus, the role of emergency care in managing these cases is increasing.

There is a current need for guidelines on the emergent management of first-time cancer-diagnosed patients [7]. Up to a third of cancer-related emergency visits in developed countries are for previously undiagnosed cases [8]. Studies have shown that patients diagnosed with cancer following an ED visit are more advanced, have poorer outcomes, lower survival rates, and are less likely to receive therapeutic care with curative intent, often independent of the cancer stage [9,10]. Pinpointing these de novo patients with high risk for adverse events is detrimental to developing improved care plans to reduce unfavorable outcomes, improve treatment accessibility, and reduce mortality.

Oncological emergencies are associated with significant morbidity and mortality. These can be defined as acute conditions caused by neoplasia or its treatment, which require rapid intervention to avoid complications or death [11,12]. Cancer often occurs in the elderly population, which has high levels of comorbidity and frailty that predispose them to treatment complications, making them particularly vulnerable to life-threatening conditions and death [13,14]. These patients often develop conditions that require emergent management, such as acute respiratory failure, severe pain, mechanical obstruction due to tumor growth, metabolic disorders, jaundice, hemorrhage, anemia, fever, infection, dehydration, nausea, and vomiting, or various other toxic effects generated by treatments [12,15,16]. Fortunately, many of these emergencies are preventable (especially the metabolic ones), as they often occur following oncological therapy [17]. An appropriate evaluation is essential for all patients with oncological emergencies. Furthermore, emergency care specialists are frequently the first evaluators of oncological patients before any oncological consult, and their role in patient outcomes is often significant as they address complications associated with the cancer diagnosis [17,18].

Several studies have explored the demographic characteristics and care needs of patients presenting to the ED with cancer and identified a high burden of first-time diagnoses on emergency care, particularly in Asia, the United States, and Canada [8,9,19]. However, there is an evident lack of data concerning Western Europe. In light of the global upsurge in daily oncological diagnoses and the associated emergency symptoms, we proposed a study focusing on oncological emergencies at the largest emergency hospital in northeastern Romania. This study aimed to identify the burden of end-stage and first-time diagnoses on emergency care. Our objective was to describe the demographic characteristics of patients requesting emergent healthcare for cancer-related symptoms and to identify their care needs. Ultimately, we aimed to identify potential interventions to alleviate the burden of cancer-related presentations on emergency services.

## 2. Materials and Methods

### 2.1. Study Design and Setting

We conducted a retrospective study, including patients diagnosed with neoplasms who received emergency medical care at the Emergency Department of the “Sf. Spiridon” County Emergency Care Hospital in Iași, Romania, between 1 October 2022 and 30 September 2023. Our hospital is the largest emergency hospital in the northeast region of Romania and the only emergency hospital in the county, receiving all emergency care requests from the city of Iasi, the county, and the most severe cases from the country’s northeast region. The hospital contains all relevant clinical and surgical departments, except gynecology, urology, oncology, and neurosurgery, with specialized hospitals available in the city for these pathologies. All cases are first evaluated in the emergency department of our hospital and then redistributed to these hospitals accordingly.

### 2.2. Patient Cohort

The inclusion criteria were as follows: patients aged 18 years or older admitted to the emergency department during the specified period with a known diagnosis of neoplasm or a suspected diagnosis based on clinical or paraclinical findings confirmed in the emergency department.

### 2.3. Data Collection

Data were retrieved from emergency care registries and electronic data records of the emergency hospital. Trained nurses record data in emergency care registries and electronic records. Emergency care residents and the research team collected demographic data, oncologic history, and patient care data from these records. We reviewed all emergency care visits within the studied interval to identify patients presenting with a cancer diagnosis or symptoms highly suggestive of cancer (alarm symptoms). Only patients who had a confirmed diagnosis or were further confirmed positive for cancer following hospital admission or other diagnostic procedures were included.

### 2.4. Statistical Analyses

Data analysis was performed using SPSS version 23 (IBM Corp., Armonk, NY, USA). Descriptive statistics were calculated for all variables. Inferential statistics, including independent samples *t*-tests (after Levene’s test for homogeneity of variances) and chi-square or Fisher’s exact tests (as appropriate), were used to compare means and frequencies between groups. Risk analysis was conducted to assess the association between the moment of diagnosis (first-time cancer diagnosis, known diagnosis) and stage at diagnosis (end-stage or other stages) and specific care needs (need for medical care, surgical care, specialty consult, intensive care, intubation and mechanical ventilation, ward admission, local hospital transfer, or out-of-county hospital transfer, and revisits to the ED). Other emergency care outcomes assessed included home discharge with recommendations or death, with odds ratios (OR) and 95% confidence intervals (CI) reported. Missing data were recorded for some variables, and cases containing missing data were removed from the respective analysis. A significance level of α = 0.05 was considered statistically significant.

### 2.5. Ethics

This study was approved by the Ethics Committee of “St. Spiridon” Emergency Hospital (approval number 114/2024). Given the retrospective nature of the study, informed consent from patients was waived.

## 3. Results

### 3.1. Sample Characteristics

**Figure 1:**
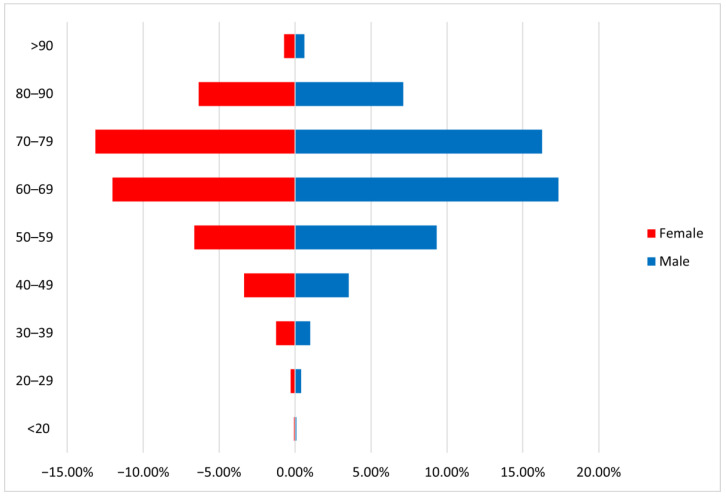
Age distribution among emergency patients with a cancer diagnosis.

**Figure 2:**
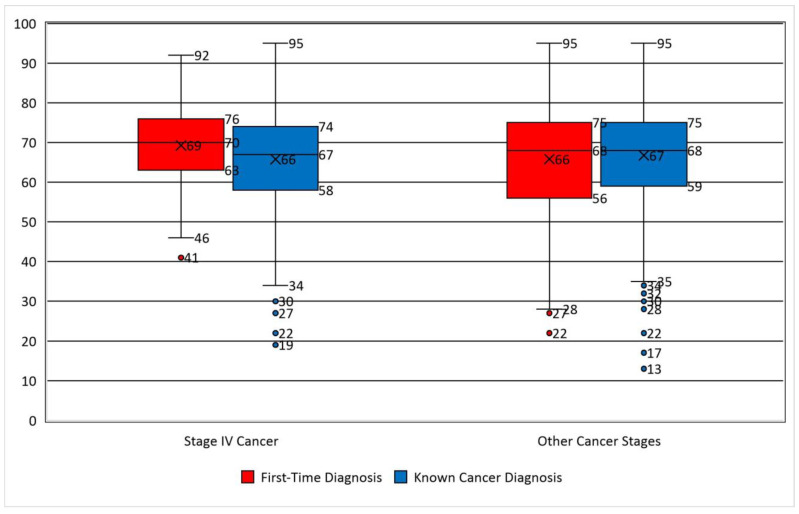
Age difference between patients with a known and first-time cancer diagnosis according to cancer stage.

During the analyzed period, a total of 81,276 emergency department presentations were recorded, of which 2510 were for oncological patients (Cancer Patient Presentation Rate-PPR: 3.08%). After adjusting for repeated presentations, we identified 193 records of recurring visits to the ED of the same individuals. Thus, our final cohort included 2318 unique patients: 1294 men (55.82%) and 1024 women (44.18%). Missing data for age was recorded in 10 cases, and these were removed from all age-related analyses. The average age in the study group was 66.43 years, with no statistical difference between genders. The most frequent age group was 70–79 years (29.42%), closely followed by the 60–69 age group (29.38%). Only 3.1% of patients were in the younger age group of 18–39 years (Figure 1). Among young patients, the most frequent gender was female (1.6% female vs. 1.42% male). There was no statistical difference in age between people with a known diagnosis and a first-time cancer diagnosis overall (mean age 65.44 vs. 66.34, *p* = 0.86); however, patients with a first-time diagnosis and end-stage cancer were older than patients with a known end-stage cancer diagnosis (mean age 69.27 vs. 65.79 for first-time diagnosis and known cancer, *p*-0.02) (Figure 2).

**Figure 3:**
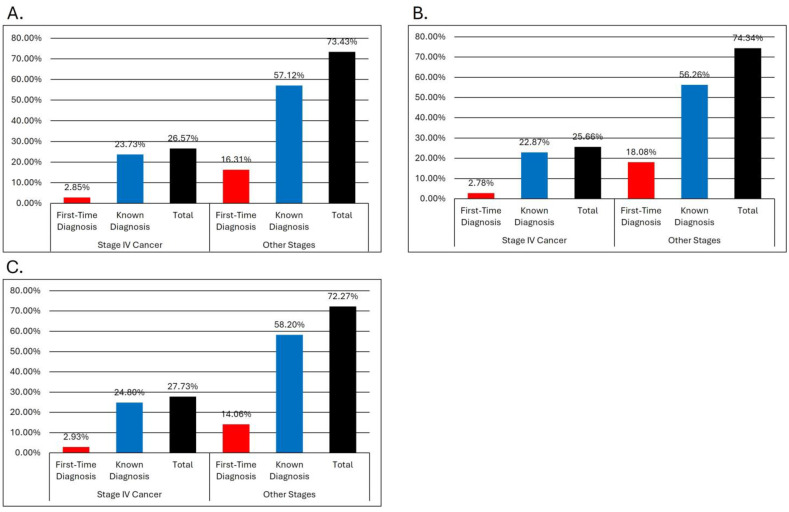
Case distribution according to stage and moment of diagnosis: (**A**) overall; (**B**) as a percentage of the male group; (**C**) as a percentage of the female group.

A total of 1874 patients (80.85%) had a previous cancer diagnosis, while 444 patients (19.15%) presented with a high suspicion of neoplasia during their initial emergency department visit, a diagnosis subsequently confirmed by diagnostic tests after a median latency period of 2.5 days. A first diagnosis was associated with gender male (270 male vs. 174 female, *p* = 0.02). A total of 26.57% (616) of patients had end-stage cancer, with the most frequent locations of metastasis being liver metastases, lung metastases, and lymph node metastases. Of these, only 2.85% (66) were for patients with a previously unknown diagnosis. There was no difference between genders regarding the proportion of de novo diagnosis in the end-stage group, but there was a significant difference in other stages, with the male gender having higher proportional rates of newly diagnosed cases (18.08% for males vs. 14.6% for females, *p* = 0.017) (Figure 3).

The statistical analysis of cancer types identified the most frequent sites as digestive (40.29%), respiratory (21.05%), and reproductive (21.05%), respectively (Table 1). The most common cancer types in the analyzed study group were colorectal cancer (18.46%), bronchopulmonary cancer (14.58%), and breast cancer (7.77%) (Supplementary Figure S1). Male gender was strongly correlated with respiratory, digestive, urinary, and musculoskeletal neoplasia (*p* < 0.001 for respiratory and digestive and *p*-0.02 for urinary and musculoskeletal, respectively). The most frequent locations for men were the lung, colon, and prostate (9.53%, 7.46%, and 4.83%, respectively). Female gender was strongly correlated with reproductive, endocrine, and other cancer categories (*p* < 001, *p* = 0.001, and *p* = 0.04, respectively), and the most frequent locations were breast, colon, and lung (7.64%, 5.18%, and 5.05%, respectively). There was a disparity in cancer type between patients with a known and a first-time diagnosis of cancer (*p* < 0.001). The most common neoplasms diagnosed in this category were lung (3.84%), colon (2.55%), and gastric (1.86%), as detailed in Supplementary Figure S2.

Most patients were monitored and treated in the emergency department and then admitted to relevant clinical wards or discharged home with therapeutic recommendations. A total of 193 patients (6.64%) had multiple ED visits, averaging 2.27 (±0.75) visits, which correlated with the female gender (*p* = 0.04). Male gender correlated with local hospital transfer (*p* = 0.03), most likely because of the high rate of urinary cancers that were transferred to the local nephrology hospital. A first-time diagnosis of cancer in the emergency department strongly correlated with the need for medical care, specialty consult, and hospital admission (*p* < 0.001 for all). Patients with stage IV cancer had, on average, more days of observation (0.86 vs. 0.13 days, *p* < 0.001) and a higher number of ER visits (1.13 visits for end-stage vs. 1.06 for other stages, *p* < 0.001). In-hospital mortality within the emergency department was low for cancer patients, at 0.95%, strongly correlated with end-stage patients (*p* = 0.01). There was no in-hospital death of first-time diagnosed patients.

### 3.2. Risk Analyses

Risk analysis detailed in Table 2 shows that patients with a de novo cancer diagnosis were more likely to be admitted to a ward, require a specialty consultation, or need any form of medical care (OR 2.09, 95% CI: 1.70–2.59, *p* < 0.001; OR 3.28, 95% CI: 2.48–4.35, *p* < 0.001; OR 2.65, 95% CI: 2.12–3.32, *p* < 0.001, respectively). Consequently, these cases were less likely to be discharged home without further care (OR 0.33, 95% CI: 0.25–0.44, *p* < 0.001). However, they were less severe, as none required intensive care and were less likely to need intubation and assisted ventilation (OR 0.36, 95% CI: 0.14–0.89, *p* = 0.02). First cancer diagnosis and end-stage disease did not overlap, as most patients did not have metastasis when the emergent care occurred (OR-0.42, 95% CI: 0.32–0.56; *p* < 0.001). End-stage cancer patients had higher odds of having repeated ER visits (OR 1.85, 95% CI: 1.32–2.59, *p* = 0.001) and a more severe condition (requiring monitoring, transfer to another hospital, intensive care, intubation, and higher mortality risk).

## 4. Discussion

The growing number of oncological patients requesting emergency care at all stages and moments of care, whether at the onset of disease, moment of diagnosis, during treatment, or at the end-of-life stage, emphasizes the crucial role of emergency departments in cancer care. There is a lack of literature data describing the burden of oncological emergencies, in general, and, in particular, the load of new diagnoses and end-stage care in emergency settings. Our study was undertaken in response to the increasing demand for emergency services among cancer patients and the increased need to improve and integrate all aspects of oncology care in emergency departments. By focusing on oncological emergencies in the largest emergency county hospital in the northeastern region of Romania, we aimed to fill the gap in the literature about the demographic characteristics and care needs of cancer patients with end-stage disease or a first-time diagnosis presenting to the ED in Western Europe.

Our study found that a representative cohort of patients requested emergency medical care for cancer-related symptoms (2318), of which 19.15% presented with a de novo cancer diagnostic, and 26.57% were end-stage cancers. Expectedly, end-stage patients were more severe and had increased odds of needing intensive care treatment (OR 4.63, *p* = 0.04), while first-time diagnosed patients required further assessment in a specialty department and were more often admitted to a ward. Cancer patients may present to the ED at any stage of the cancer survivorship continuum: at diagnosis, during treatment, post-treatment, or at the end of life [20]. Studies show that many of these emergency room presentations are preventable through appropriate population interventions and primary care [10]. Several situations can be highlighted in which the role of care is crucial: (1) the neoplasm is diagnosed incidentally while the patient is being investigated for unrelated conditions that led them to seek medical assistance (such as vehicle collisions, domestic accidents); (2) acute manifestations of neoplastic disease, known or unknown, requiring emergency care; (3) pathway used by patients without medical insurance or those who request medical care before their scheduled appointment with the attending oncologist (as an entry point for hospital admission).

Cancer patients who seek medical assistance in the ED, compared to those who receive a diagnosis in other settings, have been shown to have higher comorbidity, present with more advanced-stage cancers, and have a poorer prognosis [7,10]. A non-negligible amount of our studied group was diagnosed with cancer for the first time during their ED visit, aligning with the specialized literature indicating that 12–32% of cancer diagnoses occur in the ED [20]. However, in our cohort, the majority of de novo cases were not severe and were not in the advanced stage, suggesting a lack of proper primary care management of cancer cases in our region. Another recent study suggests that first-time diagnosis of cancer in the ED varies according to the geographic region investigated, as a large cohort study evaluating 14 regions across six countries with up to 857,068 emergent cancer presentations found de novo cancer presentation rates between 24% and 45.2% [21]. In the US, a SEER (Surveillance, Epidemiology, and End Results Program) database analysis found that of the total 614,748 cancer cases registered between 2008 and 2017, 23% were diagnosed as a result of emergency care requests [19]. In Canada, up to a third of cancer emergency department presentations were de novo cases [8]. However, these studies considered a limited number of cancers [10,19,21]. We performed a robust examination of ED involvement in diagnosing all cancer types. Based on our findings, the de novo cases were usually not severe and were more likely to need specialty consults and be admitted to a ward. A recent article by Hong et al. found that self-referred cancer patients to the ED did not require admission to a specialty ward following emergency department visits more than patients referred by doctors [22]. This emphasizes the increasing role that emergency care plays in cancer diagnosis in our region. Diagnosing cancer in the ED, whether incidental or due to specific symptomatology, is becoming an increasingly important method. Thus, emergency specialists face significant adversities in managing these cases and could benefit from multidisciplinary assessments, such as in situ oncological consultations and palliative care assessments.

The distribution of cases according to histology in our study overlaps with the global cancer epidemiology for each gender [23]. Our study found a high rate of addressability for patients with lung, breast, and colorectal cancers. This aligns with previous research that found similar distribution by cancer type [24,25]. Because these statistics largely reflect the prevalence of surviving patients diagnosed with cancer and may not account for patient-specific information, it is difficult to affirm that specific cancers require more emergent care. Efforts have been made, though, to identify factors influencing emergency care needs (like comorbidity, chemotherapy, and radiotherapy), such as in the case of patients with colorectal cancers [26]. The symptoms prompting ED visits may be directly cancer-related, or the diagnosis may be incidental. In either case, it is challenging to determine if these ED presentations could have been avoided through different triage codes or improved primary care/cancer screening access. Understanding how cancer patients present to the ED for diagnosis is crucial for improving their care and follow-up. The relationship between increased ED-diagnosed cancers and limited access to primary care or cancer screening remains unclear [7]. A recent study has shown that older people, patients with low education, and low income were more likely to be first diagnosed through emergency care [2]. However, data such as education and income level were unavailable in our registries. Regarding first-time diagnosed patients, we found that patients with de novo cancer diagnosis and end-stage cancer are older compared to patients with a known cancer diagnosis, which suggests a lack of epidemiologic surveillance of this vulnerable cohort at a population level or limited access to healthcare. We also found a significant difference in cancer localization distribution (*p* < 0.001). The largest proportion of these cases was still diagnosed with lung, colon, and gastric cancers (20.05%, 13.29%, and 9.68%, respectively). The reduced rates of reproductive de novo cancer highlight the critical impact populational screening programs have at a national level. Romania does not have a functional lung cancer screening program set up yet, while colorectal cancer screening is still in its infancy, which might explain these rates [27]. Similar findings have been reported in Taiwan following an extensive five-year multicentric retrospective analysis of 389,043 cancer cases presented to ED for medical care [10]. In this study, from the 59,423 (15.3%) first-time diagnoses, most were for lung cancer (25.1%) and colorectal cancer (23.6%), while breast cancer represented just 2.8%, suggesting the importance of community screening actions in reducing first-time cancer diagnostic burden on emergency care. Screening has significantly modified the histologic distribution of first-time cancer diagnosis in developed countries, as McPhail et al.’s study on six countries found pancreatic cancer the most often first-time emergent cancer diagnostic (34.1% to 60.4%), while the lowest rates were for colorectal cancers (9.15 to 19.8%) [21]. These results are influenced by the level of populational awareness and public perception of cancer, healthcare system development, and screening program access.

Our study found that 3.08% of all ED presentations during the analyzed period were oncological patients. While this percentage may seem insignificant, it represents a substantial number of visits to emergency departments already overwhelmed by various ailments. These percentages are comparable to the proportion of visits due to other conditions such as congestive heart failure (4%), chronic kidney disease (3.5%), cerebrovascular diseases, including stroke (3.7%), and widespread chronic conditions like diabetes (6%) [28]. A recent study on a large national database (NEDS) on emergency room presentation with cancer in the United States for the interval 2006–2012 found a presentation rate of 4.2% [24]. The study also shows that cancer patients were more likely to have an ER visit within the first year of diagnosis and have repeated visits. The difference in the observed rate of cancer presentations is most likely population-specific and because of the time period assessed. Novel therapeutic interventions and quality of care have since significantly improved patient outcomes [23]. A previous assessment by Cimpoesu et al. of the same issue in our emergency department for the assessed period of June 2009 and May 2010 revealed that oncological patients accounted for 3.26% of total presentations [29]. Although this presentation rate is similar, and only a slight reduction can be observed, in our assessment, we reported this rate to the 81,276 presentations while they reported this rate to the 40,322 presentations within that period, with only 1315 cases of emergencies for oncologic patients. There has been a significant increase in ED overcrowding, which is projected to be an upward trend [30]. As the prevalence of neoplasia increases, more and more patients will request emergent aid. Comparatively, our study further shows a reduction in the presentation rate of end-stage cases, as the survey above reported up to 67.07% of patients having metastasis, while their observed de novo case rate was similar (23.12%). The constant rate of de novo cases diagnosed in the ED is concerning and raises questions about screening programs’ efficiency in preventing emergent care needs for cancer patients. These findings highlight that requesting emergency healthcare is sometimes unavoidable for oncological patients. The observed rates can help project healthcare needs and resource utilization for this patient population. Thus, further studies are needed to identify these needs and provide evidence of improved care and patient outcomes in the emergency department.

The elderly age group constitutes the majority of ED presentations for cancer in our research. This is due mainly to the demographic distribution of cancer that affects particularly the older population [31]. It is known that cancer disproportionately affects the elderly population and that this population is at risk for unfavorable outcomes. These cases are challenging because of their increased comorbidity and associated geriatric syndromes like frailty [14]. New cases diagnosed in the ED are not different in age distribution from patients with a new diagnosis (*p* = 0.68), except for the end-stage patients, who, in our cohort, were actually older. Thus, elderly patients could benefit from a multidisciplinary assessment containing a geriatric evaluation to fine-tune their care in the context of emergency care.

The literature shows that a large proportion of cancer patients have multiple ED visits [20,25,32]. A California population-based study reported that 20% of patients with cancer had one ED visit, 8% had two visits, and 7% had three or more visits within 180 days of diagnosis [25]. In our one-year retrospective cohort study, 6.64% of patients had multiple ED visits, averaging 2.27 (±0.75). End-stage patients were more likely to present repeatedly to the ED in our study. The literature shows that patients with cancer are more likely to be admitted to a progressive care or intensive care unit (11%) compared to the general population (2%) [20]. Between 34% and 49% are discharged home, and approximately 4–5% of the remaining patients are transferred to another facility, die during the ED visits, or leave before physician evaluation or against medical advice [26]. The vast majority of patients included in our study were monitored and treated urgently, and then they were hospitalized in specialized clinical departments (32.53%) or could go home with therapeutic recommendations (31.79%). The low hospitalization rate highlights the importance of ambulatory palliative care and its potential to decrease ED overload.

Finally, the mortality rate of patients in the emergency department was minimal (0.95%), with the most common cancer diagnostics being lung cancer (0.17%). Expectedly, end-stage cancer patients presented high odds of mortality following emergency care (OR 4.06, 95% CI: 1.73–9.54; *p* = 0.001). In our study, there was no fatality for newly diagnosed cancers. A New Zealand study on lung cancer patients found that patients diagnosed via emergency care had significantly lower survival rates (adjusted OR-2.4, 95% CI: 2.1–2.74) [33]. An increased mortality rate was also observed in a study performed in Denmark (1.4% vs. 53% between normal diagnostic pathways and emergency care first-time diagnosis), with patients requesting emergency care for unknown previous cancers having increased odds of death (OR-3.38, 95% CI: 3.24–3.52) [34]. This means that patients without a previously known diagnosis are more vulnerable and could suffer more severe unfortunate outcomes. Yet, this does not consider variables like disease and patient characteristics that could significantly influence this result. Our study only assessed in-hospital mortality; thus, no long-term outcomes could be studied for first-time emergency care-diagnosed patients, which might explain the difference in findings.

Our study is a follow-up study on cancer epidemiology within the largest county hospital in Iasi (Romania), focusing specifically on patients with a novel cancer diagnosis and end-stage disease following emergency care. This is one of the first demographic assessments of first-time cancer diagnostics in Western Europe in a large-volume emergency care hospital. The generated data were used to assess the correlation between cancer diagnosis and care needs in the emergency department. There is a great need to improve primary care access and screening program availability in Romania (for lung, colorectal, and prostate cancer specifically), particularly for vulnerable groups. Facile access to oncology consultations and other diagnostic procedures could further reduce the burden of diagnoses on ED and improve patient outcomes. The participation of private hospitals in the care network of cancer patients with accessibility through national healthcare insurance has already been implemented to facilitate access to medical care [35]. However, improvements in patient outcomes have yet to be proven. Furthermore, there is an increased need for palliative care centers in our area and nationwide to reduce emergent healthcare requests for advanced-stage cancers. Our hospital has no oncology, geriatric, or palliative care staff available for emergency care consultations. Including these services could significantly shorten the time from diagnosis to treatment and help improve treatment plans for patients with cancer in the ED.

This study’s findings are limited by its retrospective design. Patients often present with alarm symptoms for neoplasia in the ED and are later confirmed in other clinics or departments [9]. A study on colorectal cancer shows that 20% of cancers have alarm symptoms up to a year earlier than the diagnosis [36]. Thus, some of the patients investigated in the ED with symptoms suggestive of novel cancer diagnostics were confirmed in other hospitals and clinics and were lost to this analysis. Furthermore, data in the hospital database was fairly limited, as no data about previous primary care referrals, other specialty consults, or cancer screening participation within the previous year was available. Thus, no inference about the medical care accessibility of patients with de novo cancer can be made. A high suspicion of cancer could have been ascertained by primary care, but some patients often chose the emergency care route for earlier confirmation. It is unclear how many of these first-time diagnoses could have been avoided through primary care management and cancer screening. This single-center study was developed in a large university emergency hospital that functions according to national regulations and ensures care to all who request it. However, there could be differences in demographic accessibility to primary care and screening in our region, limiting the reproducibility of our findings in other centers. We could not assess the repeated visits patients could have had in other emergency departments in the county. Furthermore, the university hospital receives severe cases more often, limiting the generalizability of our findings for smaller centers. Further studies are required to identify patient outcomes of de novo versus known cancer diagnosis in a prospective design, and additional studies should describe first-time cancer diagnosis burden in smaller emergency departments.

## 5. Conclusions

The role of the emergency department in evaluating and treating cancer patients is often underemphasized. Our study demonstrates that a significant proportion of patients receive their initial cancer diagnosis following emergency care and that emergency departments often carry the burden of care for end-stage cancer patients. While first-time diagnosed cases are typically less severe, they still demand considerable emergency room healthcare resources. In contrast, end-stage cancer patients are recurrent visitors of the ED, exhibiting variable degrees of severity that require complex care.

These findings highlight the importance of a multidisciplinary approach that fosters close collaboration between oncologists, geriatricians, palliative care specialists, and emergency care providers to ensure optimal care for cancer patients. Implementing national screening programs could further reduce the cancer diagnosis burden in emergency care. Furthermore, we show the importance of well-coordinated end-of-life care in reducing emergency department overcrowding and assert the critical role of outpatient palliative care services.

## Figures and Tables

**Table 1:** Sample characteristics and correlation with moment of diagnosis and cancer stage.

Variable	Total	Moment of Diagnosis			Cancer Stage		
		First-Time Diagnosis	Known Diagnosis	*p*	End Stage	Other Stages	*p*
Age, mean (±SD)	66.43 (± 12.93)	66.34 (±13.88)	66.45 (±12.7)	0.86	66.16 (±11.72)	66.53 (±13.35)	0.55
Gender (male)	1294 (55.82%)	270 (11.6%)	1024 (44.2%)	0.02	332 (14.32%)	962 (41.5%)	0.26
First diagnosis *n* (%)	444 (19.15%)	-	-		66 (2.85%)	378 (16.31%)	<0.001
Stage IV neoplasia	616 (26.57%)	66 (2.85%)	550 (23.72%)	<0.001		-	-
Neoplasia							
Respiratory	488 (21.05%)	117 (5%)	371 (16%)	0.002	136 (5.86%)	352 (15.18%)	0.47
Digestive	934 (40.29%)	220 (9.49%)	714 (30.8%)	<0.001	224 (9.66%)	710 (30.62%)	0.02
Reproductive	488 (21.05%)	61 (2.63%)	427 (18.42%)	<0.001	170 (7.33%)	318 (13.71%)	<0.001
Urinary	135 (5.82%)	19 (0.81%)	116 (5.0%)	0.12	38 (1.64%)	97 (4.18%)	0.67
Hematologic	125 (5.39%)	12 (0.51%)	113 (4.87%)	0.005	9 (0.39%)	116 (5%)	<0.001
Skin	51 (2.20%)	3 (0.12%)	48 (2.07%)	0.15	12 (0.52%)	39 (1.68%)	0.62
Endocrine	20 (0.86%)	1 (0.04%)	19 (0.81%)	0.1	6 (0.26%)	14 (0.6%)	0.73
Neuro-sensorial	32 (1.38%)	5 (0.21%)	27 (1.16%)	0.6	2 (0.08%)	30 (1.29%)	0.009
Musculoskeletal	11 (0.47%)	3 (0.12%)	8 (0.34%)	0.49	2 (0.08%)	9 (0.39%)	0.53%
Others	34 (1.47%)	3 (0.12%)	31 (1.33%)	0.12	17 (0.73%)	17 (0.73%)	0.002
Repeated visits	154 (6.64%)	23 (0.99%)	131 (5.65%)	0.17	60 (2.59%)	94 (4.06%)	<0.001
Home discharge	737 (31.79%)	69 (2.98%)	668 (28.84%)	<0.001	189 (8.16%)	548 (23.66%)	0.48
>24 h monitoring	32 (1.38%)	2 (0.08%)	30 (1.29%)	0.06	19 (0.82%)	13 (0.56%)	<0.001
Hospital admission	754 (32.53%)	206 (8.89%)	548 (23.64%)	<0.001	194 (8.34%)	560 (24.16%)	0.52
Other county hospital transfer	10 (0.43%)	3 (0.13%)	7 (0.3%)	0.38	3 (0.13%)	7 (0.3%)	0.73
Local hospital transfer	784 (33.82%)	153 (6.6%)	631 (27.22%)	0.75	233 (10.05%)	551 (23.77%)	0.014
Specialty consult	1587 (68.46%)	380 (16.39%)	1207 (52.07%)	<0.001	427 (18.42%)	1160 (50.04%)	0.59
Medical care	529 (22.82%)	171 (7.38%)	358 (15.44%)	<0.001	130 (5.61)	399 (17.21)	0.24
Surgery	227 (9.79%)	36 (1.55%)	191 (8.24%)	0.18	62 (2.67%)	165 (7.12%)	0.79
Intensive care	8 (0.35%)	0 (0%)	8 (0.35%)	0.17	5 (0.22%)	3 (0.13%)	0.35
Intubation	63 (2.72%)	5 (0.22%)	58 (2.5%)	0.02	30 (1.29%)	33 (1.42%)	<0.001
Mortality	22 (0.95%)	0 (0%)	22 (0.95%)	0.02	13 (0.56%)	9 (0.39%)	0.01
Total	2318 (100%)	444 (19.15%)	1874 (80.85%)		616 (26.57%)	1702 (73.43%)	

**Table 2:** Risk analysis for cancer stage, moment of diagnosis, and need for emergency healthcare.

	First-Time Cancer Diagnosis		End-Stage Cancer	
	OR (95% CI)	*p*	OR (95% CI)	*p*
Repeated ER visits	0.73 (0.46–1.15)	0.20	1.85 (1.32–2.59)	0.001
Home discharge	0.33 (0.25–0.44)	<0.001	0.93 (0.76–1.14)	0.51
>24 h monitoring	0.28 (0.07–1.17)	0.07	4.14 (2.03–8.42)	<0.001
Hospital admission	2.09 (1.70–2.59)	<0.001	0.94 (0.77–1.14)	0.55
Other county hospital transfer	1.81 (0.47–7.04)	0.42	1.19 (0.31–4.60)	0.73
Local hospital transfer	1.04 (0.83–1.29)	0.78	1.27 (1.05–1.54)	0.02
Specialty consult	3.28 (2.48–4.35)	<0.001	1.06 (0.87–1.29)	0.61
Need of surgery	0.78 (0.54–1.13)	0.21	1.04 (0.77–1.42)	0.81
Medical care	2.65 (2.12–3.32)	<0.001	0.87 (0.70–1.09)	0.24
Intensive care	-	-	4.63 (1.10–19.45)	0.04
Intubation	0.36 (0.14–0.89)	0.02	2.59 (1.57–4.28)	<0.001
Mortality	-	-	4.06 (1.73–9.54)	0.001

ER—emergency room.

## Data Availability

The datasets used and/or analyzed during the current study are available from the corresponding author upon reasonable request.

## Supplementary Materials

The following supporting information can be downloaded at https://www.mdpi.com/article/10.3390/medicina61010133/s1, Figure S1: Emergency patient’s cancer diagnosis distribution; Figure S2: Cancer type distribution among first-time diagnosed patients.
