# Supplementary figures and images for: Navigating Emergency Management of Cancer Patients: A Retrospective Study on First-Time, End-Stage, and Other Established Diagnoses in a High Turnover Emergency County Hospital
Source: Medicina (Kaunas). 2025 Jan 15;61(1):133. doi: 10.3390/medicina61010133 (PMC11767032; doi:10.3390/medicina61010133)

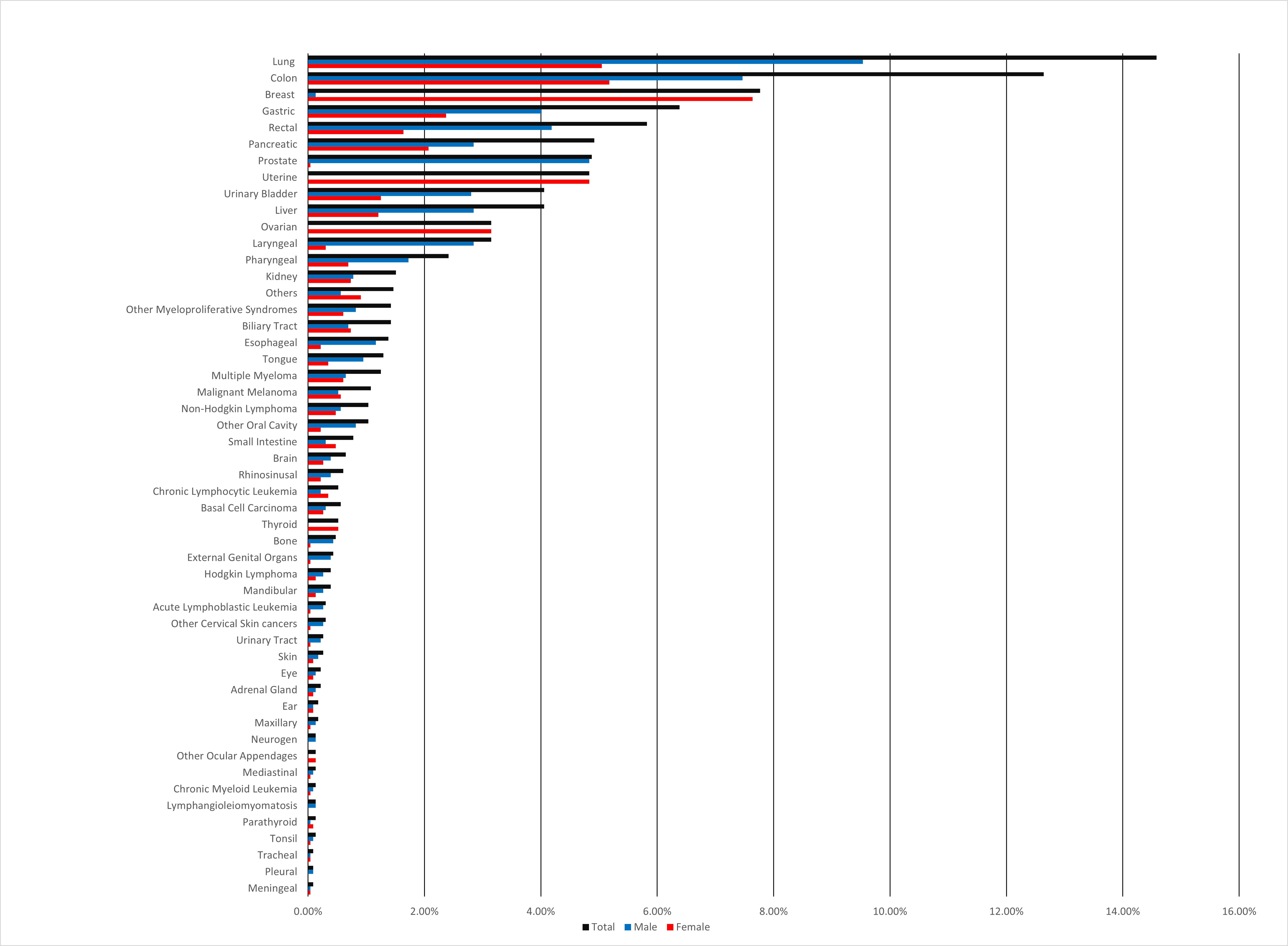

Supplement: Supplementary file 1 [file medicina-61-00133-s001.zip › supplementary files/Supplementary Figure S1.jpg]

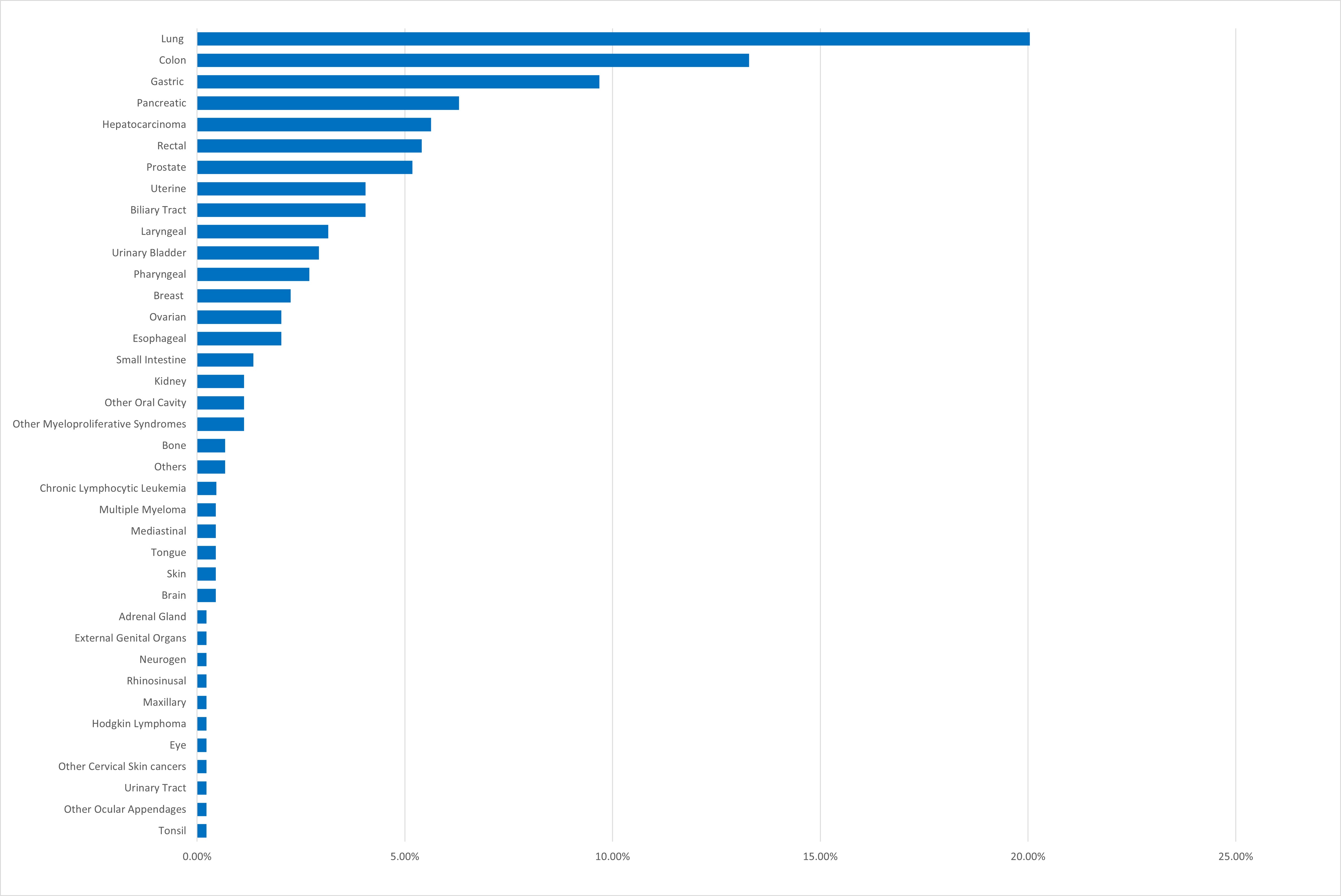

Supplement: Supplementary file 1 [file medicina-61-00133-s001.zip › supplementary files/supplementary figure S2.jpg]
